# Probing Single-Cell Macrophage Polarization and Heterogeneity Using Thermo-Reversible Hydrogels in Droplet-Based Microfluidics

**DOI:** 10.3389/fbioe.2021.715408

**Published:** 2021-10-14

**Authors:** B. M. Tiemeijer, M. W. D. Sweep, J. J. F. Sleeboom, K. J. Steps, J. F. van Sprang, P. De Almeida, R. Hammink, P. H. J. Kouwer, A. I. P. M. Smits, J. Tel

**Affiliations:** ^1^ Laboratory of Immunoengineering, Department of Biomedical Engineering, Eindhoven University of Technology, Eindhoven, Netherlands; ^2^ Institute for Complex Molecular Systems, Eindhoven University of Technology, Eindhoven, Netherlands; ^3^ Microsystems, Department of Mechanical Engineering, Eindhoven University of Technology, Eindhoven, Netherlands; ^4^ Laboratory of Soft Tissue Engineering and Biomechanics, Department Biomedical Engineering, Eindhoven University of Technology, Eindhoven, Netherlands; ^5^ Laboratory of Chemical Biology, Department of Biomedical Engineering, Eindhoven University of Technology, Eindhoven, Netherlands; ^6^ Department of System Chemistry, Institute for Molecules and Materials, Radboud University, Nijmegen, Netherlands; ^7^ Department of Tumor Immunology, Radboud Institute for Molecular Life Sciences, Radboud University Medical Center, Nijmegen, Netherlands; ^8^ Oncode Institute, Radboud University Medical Center, Nijmegen, Netherlands

**Keywords:** droplet microfluidics, single-cell analysis, cellular heterogeneity, cytokine secretion, flow cytometry, inflammation, polyisocyanide, high-throughput

## Abstract

Human immune cells intrinsically exist as heterogenous populations. To understand cellular heterogeneity, both cell culture and analysis should be executed with single-cell resolution to eliminate juxtacrine and paracrine interactions, as these can lead to a homogenized cell response, obscuring unique cellular behavior. Droplet microfluidics has emerged as a potent tool to culture and stimulate single cells at high throughput. However, when studying adherent cells at single-cell level, it is imperative to provide a substrate for the cells to adhere to, as suspension culture conditions can negatively affect biological function and behavior. Therefore, we combined a droplet-based microfluidic platform with a thermo-reversible polyisocyanide (PIC) hydrogel, which allowed for robust droplet formation at low temperatures, whilst ensuring catalyzer-free droplet gelation and easy cell recovery after culture for downstream analysis. With this approach, we probed the heterogeneity of highly adherent human macrophages under both pro-inflammatory M1 and anti-inflammatory M2 polarization conditions. We showed that co-encapsulation of multiple cells enhanced cell polarization compared to single cells, indicating that cellular communication is a potent driver of macrophage polarization. Additionally, we highlight that culturing single macrophages in PIC hydrogel droplets displayed higher cell viability and enhanced M2 polarization compared to single macrophages cultured in suspension. Remarkably, combining phenotypical and functional analysis on single cultured macrophages revealed a subset of cells in a persistent M1 state, which were undetectable in conventional bulk cultures. Taken together, combining droplet-based microfluidics with hydrogels is a versatile and powerful tool to study the biological function of adherent cell types at single-cell resolution with high throughput.

## Introduction

Studying the human immune system by conventional techniques is deeply rooted in modern cell biological research. These techniques mostly measure bulk responses of cell populations, masking functional cellular heterogeneity. To overcome this, the paradigm of cell research has shifted towards measuring with single-cell resolution, aiming to detect the smallest differences between individual cells in a population (Altschuler and Wu, 2010; Chattopadhyay et al., 2014). Measuring at single-cell level enables the identification of unique sub-populations, which are omnipresent in the human immune system (Satija and Shalek, 2014). Primary cells have a high degree of heterogeneity *in vivo*, as many stimuli gradients and physical compartments create small niches (Oyler-Yaniv et al., 2017). In bulk cell cultures, heterogeneous cellular behavior is dominantly influenced by juxtacrine and paracrine interactions, which can lead to homogenized bulk cell behavior. To retain heterogeneity, it is imperative to physically and chemically separate cells from each other during stimulation, especially in highly secretory and plastic primary cells. The field of microfluidics has yielded promising opportunities for such an approach as current research is focused on achieving single-cell resolution (Sinha et al., 2018; Luo et al., 2019).

Microfabrication of microfluidic devices using polydimethylsiloxane (PDMS) soft lithography has enabled easy access to various methods of manipulating fluids at the micro- to nanoscale (Xia and Whitesides, 1998). A promising approach for single-cell manipulation and culture is droplet-based microfluidics which allows for automated and high-throughput encapsulation of single cells in water-in-oil emulsions (Wimmers et al., 2018; Matuła et al., 2020; Ou et al., 2021). This approach has several advantages compared to other microfluidic single-cell techniques, such as microwells (Sinha et al., 2018). Fluorinated oil and surfactants ensure gas exchange, which is necessary for proper cell metabolism, and droplet stability, allowing droplet storage over prolonged periods of time (Sklodowska and Jakiela, 2017). Furthermore, cell encapsulation follows the Poisson distribution, which allows a certain degree of control over the number of cells per droplet (Collins et al., 2015). Most importantly, after single-cell encapsulation and stimulation, cells are easily retrievable for downstream analysis (Wimmers et al., 2018). Although this approach has proven successful for studying cells in suspension, there are inherent challenges when studying the behavior of adherent cells. Specifically, studying adherent cells under prolonged suspension culture conditions generates biased and biologically irrelevant findings as this might initiate anoikis-like mechanisms (Taddei et al., 2012) or affect cellular responses (Jain et al., 2019).

To tackle this challenge, several groups have incorporated hydrogels in their droplet-based microfluidic protocols (Karoubi et al., 2009; Mayfield et al., 2014). Two major advantages of hydrogels are that they provide relevant mechanical cues and allow for cell adherence (Huang et al., 2017; Mohamed et al., 2019). Commonly used in droplet microfluidics are the natural occurring alginate-based (Leong et al., 2016; Huang et al., 2017; Mao et al., 2019) or synthetic polyethylene glycol-based (PEG) (Krutkramelis et al., 2016; Kim et al., 2019) hydrogels which are primarily gelated using chemical or physical crosslinking. Although these techniques are well developed and understood addition of gelation agents has the potential to negatively affect cell phenotype and viability. Additionally, gelation is difficult or impossible to reverse which prevents cell retrieval from droplets for subsequent analysis. Thermo-responsive hydrogels such as agarose (Leng et al., 2010; Tumarkin et al., 2011) and collagen-based (Dolega et al., 2015; Tomasi et al., 2020) hydrogels are therefore a better option as they gelate based on temperature. Agarose is commonly used but not suitable as gelation is achieved by cooling. And whilst collagen-based hydrogels are commercially available and have an ideal gelation temperature at 37°C, reversing gelation is a very slow process or requires additional reagents. A promising alternative are polyisocyanide (PIC) hydrogels, which have a gelation temperature of 15°C, allowing for cell preparation at lower temperature and hydrogel formation at cell culture temperatures. Additionally, gelation is reversed quickly by cooling back down, allowing for easy cell recovery (Kouwer et al., 2013).

We previously reported on a droplet-based single-cell technology platform suitable for cells in suspension (Wimmers et al., 2018; Sinha et al., 2019). Here we aim to adapt that approach to become suitable for adherent cell types. To achieve this, we incorporated the thermo-reversible PIC hydrogel into our microfluidics platform. PIC gels exhibit mechanical properties that are comparable to the extracellular matrix, including strain-stiffening behavior (Das et al., 2016). To enhance biocompatibility, PIC gels are routinely functionalized with the cell-adhesive peptide Gly-Arg-Gly-Asp-Ser (GRGDS) (Liu et al., 2019). To validate the incorporation of hydrogel into single-cell droplets, we aimed to study the polarization of primary human monocyte-derived macrophages in the single-cell microfluidic platform. This regulatory immune cell is found in almost every part of the human body and plays an important role in maintaining homeostasis (Wynn et al., 2013). Macrophages are well known for their heterogeneous nature with phenotypes, or polarization states, ranging over a spectrum with pro-inflammatory (M1) to anti-inflammatory (M2) at the extremes (Mosser and Edwards, 2008). Furthermore, macrophages are highly secretory cells (Duque and Descoteaux, 2014) which communicate to other cells via the secretion of numerous cytokines, depending on their polarization state. To validate the microfluidic platform, we investigated the polarization potential of macrophages at the single cell level in terms of phenotype, via membrane marker expression, as well as functionality, in terms of cytokine secretion. To that end, we tested the compatibility of the PIC hydrogels with an in-droplet cytokine detection platform for single-cell TNFα detection (Wimmers et al., 2018). Both functional and phenotypical read-out were performed using flow cytometry, which has been the standard for high-throughput quantitative single-cell measurements for years (Herzenberg et al., 2002). The combined functional and phenotypical read-out allowed for the detection of cells with a persistent pro-inflammatory nature hidden in relatively anti-inflammatory cell populations. Taken together, the droplet microfluidic platform in conjunction with the thermo-reversible PIC hydrogel facilitates the discovery of heterogeneity within adherent human macrophages and is widely applicable to study adherent cell behavior at single-cell level and with high throughput.

## Materials and Methods

### Isolation and Culture of Monocytes From Human PBMCs

Macrophages were isolated from buffy coats obtained from healthy human donors (Sanquin) after written informed consent per the Declaration of Helsinki and according to the institutional guidelines. Peripheral Blood Mononuclear Cells (PBMCs) were purified from buffy coats via density gradient centrifugation using Lymphoprep (Stemcell technologies). Obtained PBMCs were plated in Nunc™ culture flasks (Thermofisher) at 1.3 × 10^6^ cells/cm^2^ in Roswell Park Memorial Institute (RPMI) 1640 (Gibco, life technologies) + 2% human serum (HS, Sanquin blood bank) + 1% Penicillin streptomycin (Gibco, life technologies). After 1 h of incubation at 37°C and 5% CO_2_ the non-adherent cells were removed, and adherent monocytes were further differentiated into macrophages.

### Human Monocyte Differentiation and Polarization

Monocytes were differentiated into macrophages for 5 days using 20 ng/ml macrophage colony-stimulating factor (M-CSF) (Peprotech) at a concentration of 1 × 10^6^ cells/ml in RPMI 1640 medium supplemented with 1% Penicillin Streptomycin and 2% HS followed by a media change on days three and six. On day six, the medium was supplemented with either 100 ng/ml interferon-gamma (IFN-y) (Peprotech) + 100 ng/ml Lipopolysaccharide (LPS) (from *Escherichia coli*, Merck) to induce a pro-inflammatory polarization (M1 state) or 40 ng/ml interleukin-4 (IL-4) + 20 ng/ml IL-13 (both Peprotech) to induce an anti-inflammatory polarization (M2 state). Cells were polarized over a period of 48 h. To detach the cells for further processing or analysis, ice cold PBS with 5 mmol EDTA (Merck) was added to the cells for 1 h at 4°C followed by gentle scraping. For the polarization in droplets, the same 6-day differentiation with M-CSF was used. Subsequently, macrophages were detached and encapsulated in droplets with media and the same cytokines as for the bulk polarization. Cytokine concentrations were adjusted to the smaller volume per cell to ensure that the mol per cell yield was identical to bulk experiments.

### PDMS Device Fabrication

Photomasks for soft photolithography were ordered from CAD/Art Services, Inc. (Bandon, Oregon). PDMS molds were produced by spin-coating wafers with SU-8 3000 photoresist (Microresist Technology’s) according to manufacturer’s protocol to obtain 30 µm of channel height. 3-inlet PDMS devices were fabricated by mixing SYLGARD® 184 PDMS with SYLGARD® 184 curing agent (both from Merck) at 10:1 w/w onto the PDMS molds and allowed to cure for 2 hours at 65°C. A 1 mm biopsy puncher was used to punch holes for the inlets and outlet. The devices were bonded to glass slides using a plasma asher (Emitech, K1050X). After bonding, the channels were treated with 5% perfluorooctyltriethoxysilane in HFE-7500 (both from Fluorochem), incubated for 1 hour at 65°C, flushed with HFE-7500, and incubated overnight at 65°C for thermal bonding.

### Production of Cooling Devices

The design for the device cooler was designed in Autocad 2020 (Autodesk®) (design is available as electronic Supplementary Material) and cut out of clear polymethylmethacrylate (PMMA) sheets using a VLS2.60DT laser cutter (Universal Laser Systems). The slides of PMMA where then attached using Acrifix acrylate glue (Evonik Industries). A glass microscopy slide was attached to the PMMA using Dowsil™ 732 silicon glue (Dow Corning) to seal of the water chamber. The pipette tip cooler was designed in Siemens NX (Siemens AG) (design is available as electronic Supplementary Material) and printed using an UP! Mini 3D printer (Tiertime). The print was smoothed out with an acetone vapor bath for 30 min. Both cooling devices were connected to a simple water pump (7026898, RS PRO) in series and ice water was used for cooling.

### Polyisocyanide Hydrogel

The azide functionalized PIC polymers were prepared as described previously (Liu et al., 2019), after which Bicyclo[6.1.0]non-4-yn-9-ylmethyl conjugated GRGDS motifs were attached to the azide groups according to previously described protocols (Das et al., 2016). Polymers were dissolved in cold medium (2 mg/ml) with or without cytokines. The cold PIC solution was inserted into the stimuli/hydrogel inlet to obtain a 1 mg/ml concentration after droplet formation.

### Production of Single-Cell Droplets and Retrieval of Cells

Droplets were generated using a tip-loading set-up, meaning that the cell and cytokine solutions were drawn into a 200 μl pipet tip and loaded to the inlets in the PDMS chip as previously described for single-cell droplets (Sinha et al., 2019). The pipet tip was connected to a neMESYS microfluidic pump (Cetoni) using tubing in which mineral oil (Merck) was used as hydraulic fluid. 2.5% PicoSurf (SphereFluidics) in HFE was flushed into the chip via the outermost inlet (at a flowrate of 30 μl/min for droplets without hydrogel and 20 μl/min when hydrogel was incorporated), while cells and the cytokines/hydrogel were flushed from the inner two inlets (at a flowrate of 5 μl/min). Cell concentration at the droplet formation point was 2.0 × 10^6^ cells/ml. For hydrogel encapsulation in droplets, a mixture of PIC and cytokines was injected into the hydrogel/stimuli inlet. For multi-cell droplets flow speeds were 10 μl/min for the oil and 1.6 μl/min for both the cells and stimuli. Droplets were collected in an Eppendorf tube from the outlet, and 150 μl of culture medium was added on top of the emulsions to prevent evaporation of HFE oil. Droplets were incubated for 48 h at 37°C and 5% CO_2_, after which they were de-emulsified by addition of 20% 1H,1H,2H,2H-perfluoro-1-octanol (PFO) in HFE-7500 at a 1:1 volume ratio. Merging of the droplets formed an interface of medium on top of HFE-7500 oil. The medium containing the cells was collected in a new Eppendorf tube and processed for further analysis.

### Characterization of Droplets; Cell Distribution and Droplet Size

After production, 2 µl of droplet suspension was added onto a glass slide and pictures were taken using an EVOS™ microscope (ThermoFisher scientific). Cell counts per droplet were counted manually to calculate the cell distribution which was compared to the predicted Poisson distribution (Collins et al., 2015). The average droplet diameter was measured from the microscopy images using ImageJ software (Rueden et al., 2017), for a minimum of 30 droplets per experiment. To determine monodispersity, the coefficient of variation was calculated. Droplets were considered monodisperse when the coefficient of variation was below 10% (S. Ten Klooster, 2019).

### Cell Staining and Flow Cytometry

In order to detect TNFα cytokine secretion, cells were coated with catch antibodies (Miltenyi Biotec) before the polarization period. After polarization, both the in-bulk and in-droplet cultured cells were obtained for staining and flow cytometric analysis. Cells were stained for viability using Zombie NIR™ (Biolegend) following the manufacturer’s protocol with a total dilution of 1:5.000 in PBS. Subsequently, cells were stained using a cocktail of fluorescent antibodies for surface markers and bound cytokines; anti-Human cluster of differentiation 80 (CD80)- APC-R700 (BD Bioscience), anti-Human C-C chemokine receptor 7 (CCR7) - Brilliant violet 421™, anti-Human CD206- PE/Cy7, anti-Human CD200R- PE/Dazzle™ 594, (all from Biolegend) and anti-human TNFα-APC (Miltenyi Biotec). Antibodies were titrated and used at a dilution of 1:40 for optimal performance. Flow cytometric measurement was performed using FACSaria III (BD bioscience) and a total of 10.000 total events were measured per sample. Results were analyzed using FlowJo (FLowJo LLC), which was also used for generating histograms and dot plots. Marker expression was quantified using Median Fluorescent Intensity (MFI). Results were plotted using Prism8 (GraphPad software).

### Confocal Microscopy

For confocal microscopy cells were incubated with Cell Tracker Green (Thermofisher) at a 1:200 dilution. 10 μg/ml of Sulfo-Cy5-amine (Lumiporbe) and Hoechst ready flow™ (Thermofisher) were added to the hydrogel solution before droplet production. Produced droplets were incubated overnight and added onto glass slides for confocal imaging using a Leica TCS SP8X (Leica Biosystems).

### Analysis of Morphology in Droplets

For analysis of macrophage morphology in hydrogel droplets ImageJ was used (Rueden et al., 2017). Using the fluorescent channel for Cell Tracker Green a binary mask was created using the automated thresholding option (Supplementary Figure 1A). Particle analysis was performed on the obtained cell shape which allowed for the calculation of morphological parameters from five basic dimensional measures; Area, Convex area, Perimeter, Major, and Minor Axis (Supplementary Figure 1B), as described below.

$$Circularity=\;\frac{4*\pi *Area}{Perimeter^{2}}$$ (1)

$$Solidity=\;\frac{Area}{Convex\;Area}$$ (2)

Circularity and Solidity describe the degree of protrusions, as cell masks of cells with more protrusions are more “frayed” which results in a higher value for the perimeter or convex area.

$$Aspect\;ratio=\frac{Major\;axis}{Minor\;Axis}$$ (3)

$$Roundness=\;\frac{4*Area}{\pi *Major\;axis^{2}}$$ (4)

Aspect ratio and Roundness describe the degree of elongation, as cell masks of cells with a more elongated shape have a larger major axis, which results in a higher Aspect ratio and lower Roundness. For each parameter a value of exactly 1 indicates a perfect circle.

### Statistical Analysis

Data are shown as mean ± standard error of the mean (SEM) or in violin plots unless indicated differently. Statistical analysis was performed using repeated-measures one-way ANOVA with post-hoc Tukey’s test after normality was proven using Shapiro-Wilk test, or with an unpaired *t* test. *p* < 0.05 was considered significant. For morphology data a one-tailed Mann-Whitney test was performed.

## Results and Discussion

To validate the droplet-based culture platform, macrophages were stimulated in suspension droplets over the course of 2 days, which resulted in the phenotypical states of pro-inflammatory M1 and anti-inflammatory M2 macrophages as also observed in bulk cultures (Figure 2). In addition, we demonstrated that cellular interactions have an influence on M2 polarization by culturing cells in multi-cell droplets in parallel with single-cell droplets (Figure 3). From that point on thermo-reversible PIC hydrogel was successfully incorporated to improve the platform for adherent macrophages (Figure 4). The addition of hydrogel resulted in an improved viability of retrieved cells and an enhanced M2 polarization (Figure 5). Additionally, by hydrogel functionalization with GRGDS we could show morphological changes of individual macrophages (Figure 6). Finally, by incorporating a cytokine catch approach we were able to show that more heterogeneity was maintained in our droplet-based platform compared to bulk culture as we observed persistent pro-inflammatory cells in non-stimulated and anti-inflammatory samples (Figure 7).

### Droplet-Based Microfluidics for High-Throughput Single Macrophage Encapsulation

**FIGURE 1:**
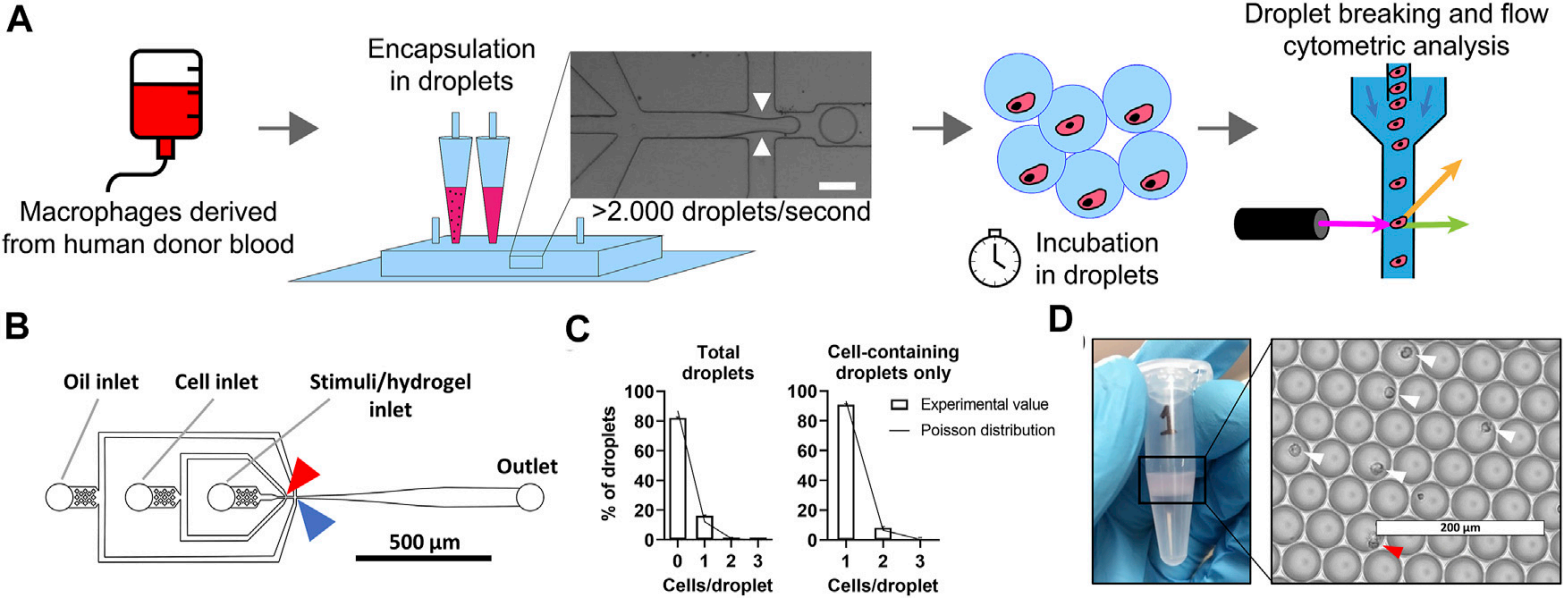
Droplet-based microfluidics for high-throughput production of identical single-cell cultures. **(A)** Schematic of workflow for single-cell culture and the single-cell analysis of primary macrophages. White arrows represent were oil cuts of droplets, scale bar is 50 μm. **(B)** To-scale layout of microfluidic chip with inlets and outlet indicated by grey lines, red arrow indicates location where cells and stimuli are combined, blue arrow indicates where droplets are formed. Channel height measured 30 μm, scale bar represents 500 μm. **(C)** Distribution of cells in droplets for both total droplets and empty droplets excluded. Bars represents data of 1,000 manually counted droplets in two independent experiments performed at a cell concentration of 2 × 10^6^ cells/ml at droplet formation point. The plotted line represents Poisson distributed values based on the same concentration. **(D)** Eppendorf tube containing one batch of 2.5 × 10^6^ droplets as collected from microfluidic device, with droplets floating on excess oil. Light microscopy picture of monodisperse droplets on glass slides, white arrows indicate single cells, red arrow indicates double cells. Scale bar equals 200 μm.

Macrophages are notoriously heterogeneous by nature and in-depth understanding of polarization of single macrophages is currently lacking. We use our droplet-based approach to compartmentalize and culture individual cells in identical microenvironments for stimulation (Figure 1A). Briefly, monocytes were isolated from healthy human donor blood and differentiated into macrophages. Next, macrophages and stimuli were kept on ice and mixed just prior to encapsulation in picolitre-sized droplets ensuring that no premature cell activation occurred (Figure 1B). As cell encapsulation in droplets follows a Poisson distribution, a certain number of droplets will be empty or contain one or multiple cells (Collins et al., 2015). We used a cell concentration of 2.0 × 10^6^ cells/ml which resulted in approximately 87% of droplets being empty and approximately 13% of droplets containing one or more cells, of which 91% contained exactly 1 cell (Figure 1C). With a production speed of ∼150.000 droplets per minute this resulted in about 2.5 million droplets produced in an 18-min sample run, of which ∼330.000 droplets contained exactly one cell. This typical experimental run is indicative for the high-throughput nature of our approach. The Poisson-predicted results corresponded tightly with experimental outcomes, verifying the predictability and tunability of the cell encapsulation, as has been commonly described in literature (Collins et al., 2015; Sinha et al., 2019). To obtain reliable single-cell cultures, reproducible monodisperse droplets are indispensable. Droplet size was homogenous (Figure 1D and Supplementary Figure 2A) with an average droplet volume of 69 ± 5.8 pl. The coefficient of variation was always well below 10%, indicating monodispersity (S. Ten Klooster, 2019 and Supplementary Figure 2A). After a period of incubation, cells were retrieved by breaking the droplet emulsions using PFO and prepared for phenotypical analysis using flow cytometry.

### Macrophage Polarization Can Be Achieved in Single-Cell Aqueous Droplets

**FIGURE 2:**
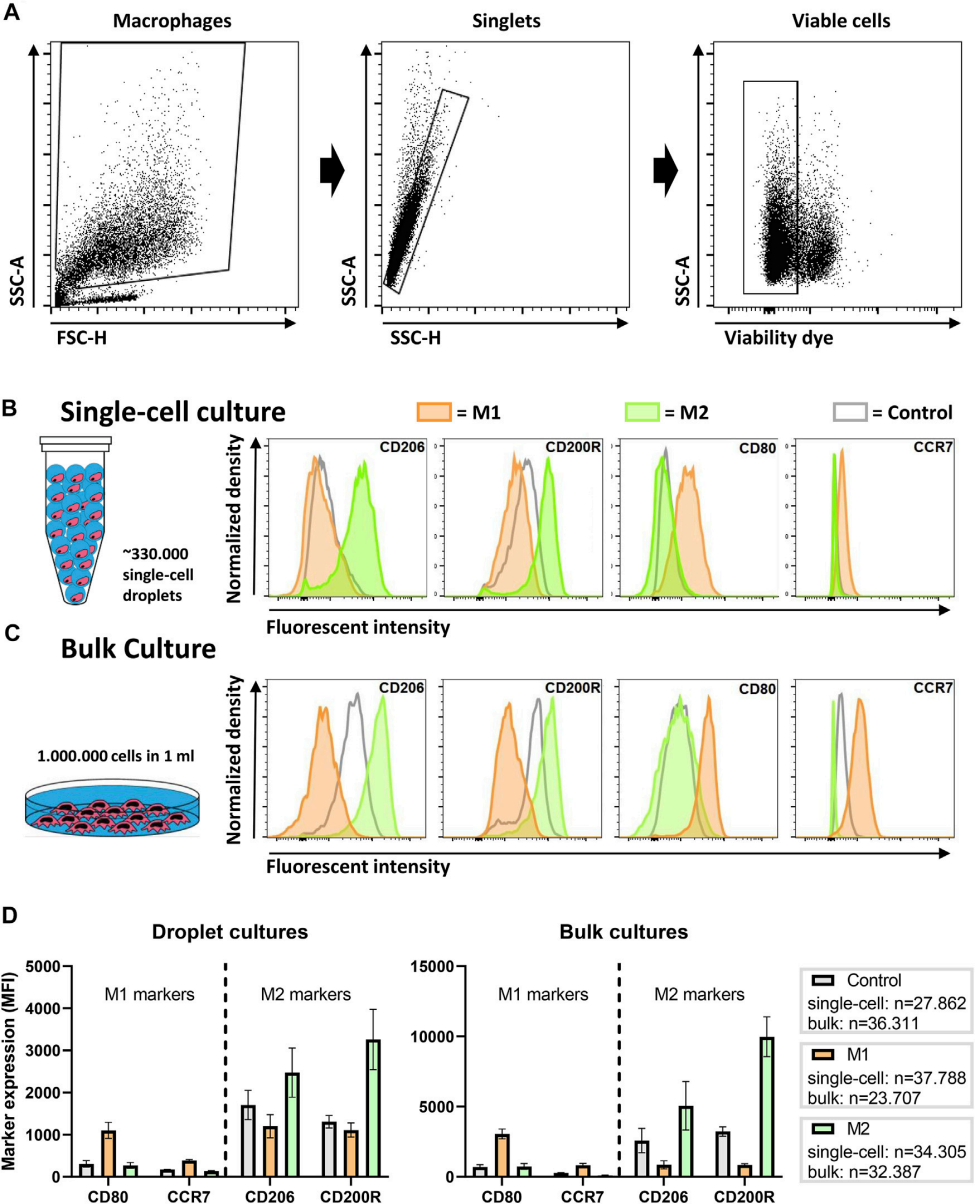
Macrophage polarization in droplets as compared to conventional bulk culture. **(A)** Gating strategy; cells are gated from debris, single cells are gated from doublets and viable cells are gated from dead cells. **(B)** Expression of single macrophages polarized for 2 days in droplets with LPS + IFN-γ (M1), IL-4 + IL-13 (M2) or as a control without additional cytokines. Histograms are taken from one representative donor. **(C**) Expression of macrophages polarized in bulk culture for 2 days with M1 and M2 cytokines or as a control without additional cytokines. Histograms are taken from one representative donor. **(D**) Bar graphs representing marker expression of M1 and M2 stimulated macrophages in bulk and droplet culture compared to control. Data represents four independent donors. “*n*” indicates number of cells measured from all donors combined.

To probe macrophage heterogeneity, we cultured and stimulated single human macrophages in picolitre-sized droplets up to 48 h. To induce distinct and widely accepted macrophage polarization states (Vogel et al., 2014), we cultured cells either with LPS and IFN-γ for a pro-inflammatory M1 phenotype, or with IL-4 and IL-13, for an anti-inflammatory M2 phenotype. These phenotypical states are widely accepted in literature as the two extremes in a much more complex spectrum of macrophage polarization (Mosser and Edwards, 2008). Thereafter, cells were recovered and, using flow cytometry, phenotypically interrogated by staining for the costimulatory molecule CD80 and the chemokine receptor CCR7, as indicators of M1 polarization, and the mannose receptor CD206 and the glycoprotein CD200R, as indicators of M2 polarization. After gating out debris, doublets and dead cells (Figure 2A), flow cytometric analysis showed an upregulation of polarization state-specific membrane markers (Figure 2B). Specifically, CD206 expression was increased for M2 stimulation conditions when compared to both the M1 stimulated and the control cells. CD200R was clearly increased compared to the M1 stimulated condition but to a lesser extent compared to the control condition. Both the surface marker expression of CD80 and CCR7 were increased after M1 stimulation and remained unchanged upon M2 stimulation when compared to the control condition. These results indicate that single-cell polarization was achieved using the droplet-based culture approach. Additionally, these results are in correspondence with similar experiments from other groups using bulk cultures, indicating that M1/M2 polarization yields two distinctly different and opposite phenotypes (Spiller et al., 2014).

To validate these single-cell results, the same experiments were performed in a conventional bulk approach with cells from matching donors. Histograms representing marker expression of bulk cultured cells display similar expression patterns as single cells cultured in droplets (Figure 2C). Interestingly, the MFI of the receptors on bulk cultured cells was considerably higher, which was also observed in control samples. The overall lower marker expression in single-cell cultures was consistently observed in independent experiments for multiple donors (Figure 2D). One obvious explanation for this difference could be the absence of cellular communication, as macrophages are highly secretory cells that easily influence each other via paracrine signaling to improve polarization (Duque and Descoteaux, 2014). In addition, the droplet-encapsulated cells are in a constant state of suspension, while macrophages are adherent cells and adhesion promotes activation (e.g., via integrins) (McWhorter et al., 2015; Cha et al., 2017). Taken together, the results show that macrophages do polarize as single cells, however, not as optimal as in conventional bulk cultures.

### Juxtacrine and Paracrine Interactions Increase the Degree of M2 Polarization When Cultured in Multi-Cell Aqueous Droplets

**FIGURE 3:**
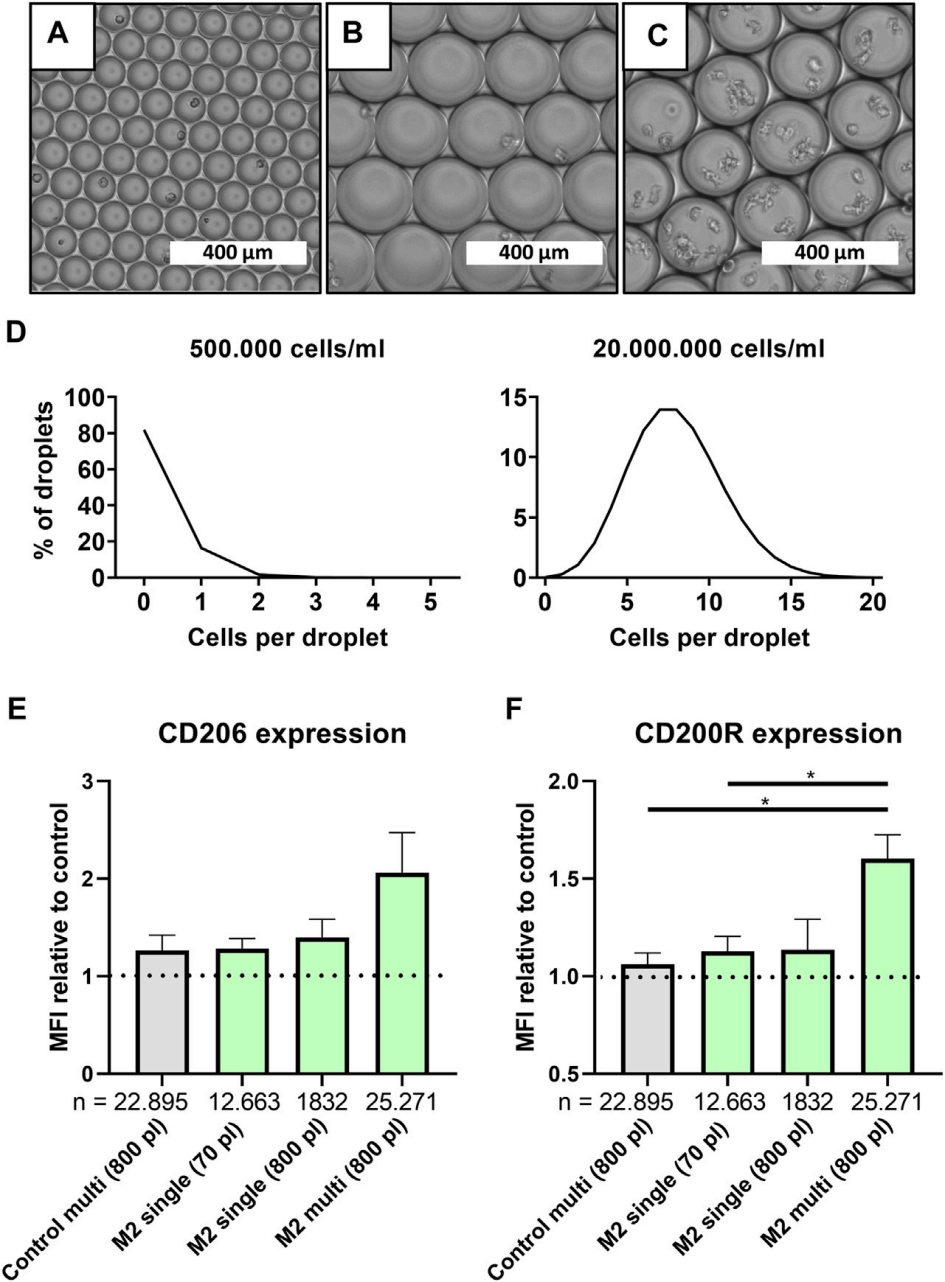
Multi-cell droplets to model cell-cell interactions in droplets. **(A–C)** Light microscopy picture of small single-cell droplets (∼70 pl), large (∼800 pl) size single-cell droplets and large multi-cell droplets, scale bars represents 400 μm. **(D)** Predicted cell distribution in ∼800 pl droplets as calculated by Poisson distribution for single-cell droplets (500.000 cells/ml) and multi cell droplets (20.000.000 cells/ml). **(E,F)** MFI relative to single-cell control samples for CD206 and CD200R, respectively, for samples cultured in 800 and 70 pl droplets with single- or multi cell encapsulation. Grey bar represents unstimulated multi-cell control and green bars represent M2 stimulated samples of three unique donors. “*n*” indicates number of cells measured from all donors combined. Statistical significance was tested using repeated-measures one-way ANOVA with post-hoc Tukey’s test where *p < 0.05. If no significance is indicated, it was not found.

Although the absence of cellular communication is generally desired in single-cell approaches, its effect on cellular function is often underestimated. To investigate the potency of cell-cell interactions on macrophage polarization at the smallest level, larger droplets were produced, which allowed for multi-cell encapsulation. This was achieved by increasing the channel height in the microfluidic device from ∼30 to ∼70 µm which allowed for stable production of droplets with a volume of 779.0 pl ± 58.7 (Figures 3A,B and Supplementary Figure 2B). Based on predictions according to the Poisson distribution, we controlled the cell concentrations required for “single-cell large droplets” and “multi-cell large droplets” (Figure 3C). For the various conditions we produced 18 min for ∼70 pl-sized droplets and 10 min for the ∼800 pL-sized droplets yielding, respectively, 330.000 droplets containing single cells and 40.000 droplets containing either 1 or ∼8 cells.

Comparing large multi-cell droplets to single-cell droplets readily showed that just the mere presence of multiple cells resulted in a slight increase of both CD206 and CD200R expression (Figures 3E,F). When M2 stimuli were also incorporated, this resulted in an even higher expression which was significant for CD200R. To control for the higher absolute number of available stimuli on a per cell level, and since confinement of macrophages has been shown to affect cell functionality (Jain and Vogel, 2018), we also encapsulated single cells in large 800 pl sized droplets. However, in our study no significant effect was observed in both CD206 and CD200R expression by increasing droplet size only. Therefore, the increased expression in multi-cell droplets compared to single-cell droplets can be attributed to an interplay between added stimuli and cellular communication, which fits the paradigm of macrophages being highly secretory cells (Duque and Descoteaux, 2014). Thus, it is likely that absence of cellular interactions plays a role in the decreased markers expression found in single-cell polarized macrophages when compared to bulk culture.

### Incorporating a Thermo-Reversible Hydrogel in Droplets for Single-Cell Adherent Culture

A prolonged state of suspension of adherent cells results in a process called anoikis, making cells more susceptible to programmed cell death (Karoubi et al., 2009; Escate et al., 2016). Moreover, the mechanosensitive nature of macrophages contributes to cellular polarization in microenvironments, where mechanical cues (Ballotta et al., 2014; Jain and Vogel, 2018) and cell shape (McWhorter et al., 2013, 2015) induce polarization. Therefore, we opted for the incorporation of a hydrogel to provide cells with a substrate for adherence and make the droplet-based single-cell culture approach more suitable for macrophages and potentially adherent cells in general.

**FIGURE 4:**
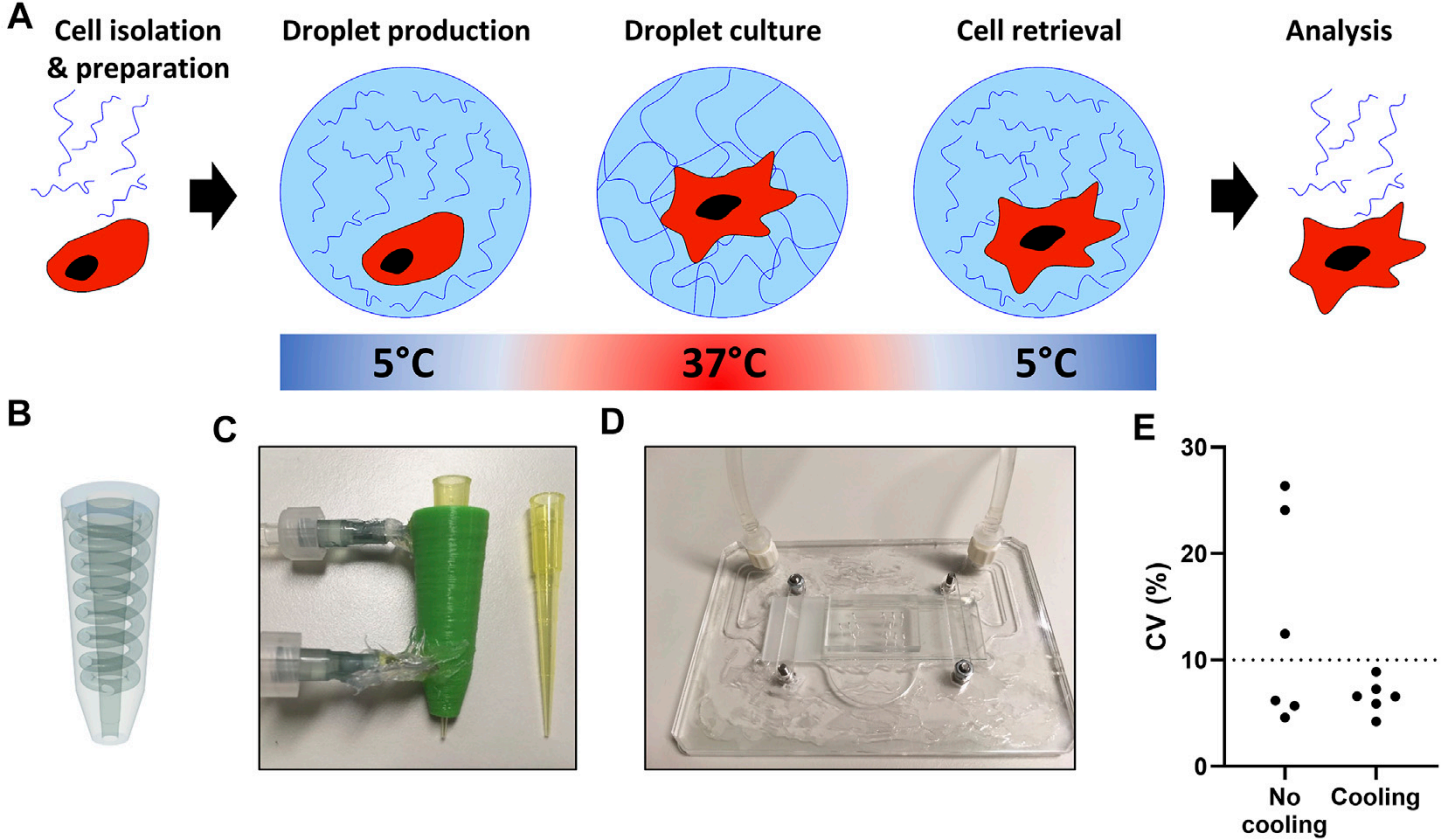
Temperature regulated production of PIC hydrogel droplets. **(A)** The hydrogel and cell encapsulation workflow; cells (red) are encapsulated with hydrogel (blue) at 5°C, as temperature increases in the cell incubator gelation occurs, after the desired culture period droplets are cooled followed by de-emulsification resulting in easy cell retrieval. **(B)** 3D design of pipette-tip cooler. **(C)** Water-cooled, 3D-printed pipette-tip cooler. **(D)** Water-cooled, transparent microscope mount, containing a microfluidic droplet device. **(E)** Coefficient of variation (CV) as calculated from *n* = 30 measured droplet sizes in six individual experiments with pictures taken from a small sample of all collected droplets per run.

A major prerequisite for our single-cell platform is that all components are biocompatible, and that cell retrieval is easy and nondestructive to allow for cellular interrogation in downstream analysis. Based on its thermo-responsive properties we used PIC based hydrogels, which are in low viscous solutions below 15°C, while gelation occurs at physiological cell culture temperatures (Figure 4A). PIC has previously been used for cell culture and can be easily functionalized with bioactive sequences, such as GRGDS, to improve adherence (Das et al., 2016; Liu et al., 2019).

To ensure droplet monodispersity and allow for easily accessible table-top droplet production, two small cooling systems were designed to cool the hydrogel during droplet production. We generated a 3D printed pipette-tip-cooler which contains the hydrogel before insertion into the microfluidic chip (Figures 4B,C), and a microscope platform which contains and cools the microfluidic device during droplet production (Figure 4D). Comparing the coefficient of variation in droplet size showed that the cooling during production was essential to achieve consistent monodisperse droplets (Figure 4E). Additionally, due to the small size and transparency of the cooling systems, droplet formation could still be monitored under the microscope during production (Supplementary Video 1).

### Macrophages Retain Viability and Show More Potent M2 Polarization When Cultured in Hydrogel Droplets

**FIGURE 5:**
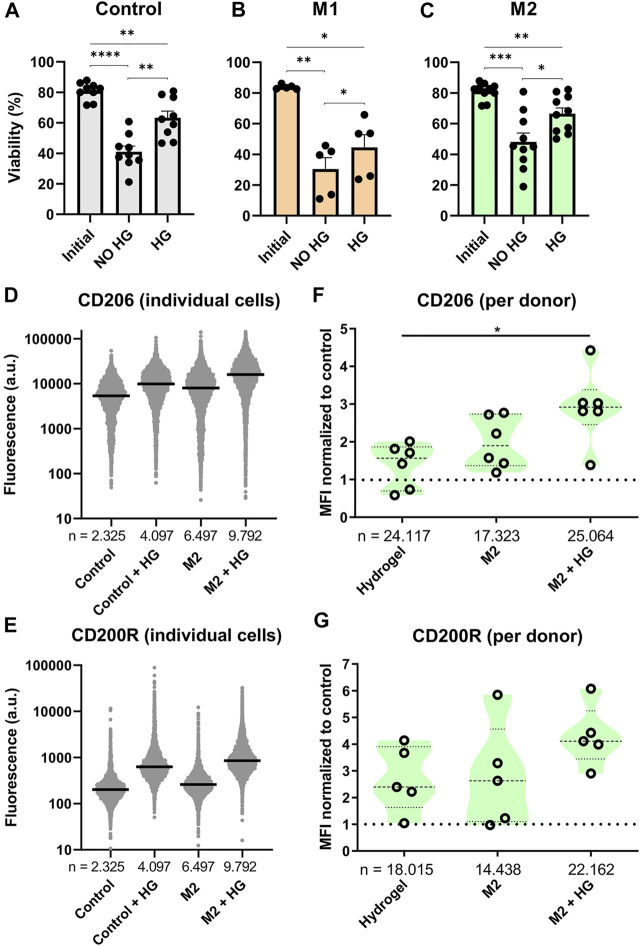
Effects of thermo-reversible hydrogel droplets on single-cell macrophage culture. **(A–C)** Viability of macrophages before (Initial) and after 2 days of single-cell polarization in droplets either with (HG) or without (NO HG) PIC hydrogel incorporated. Bars represent control (9 donors), M1 (5 donors) and M2 (10 donors) with *p < 0.05, **p < 0.01, ***p < 0.001, ****p < 0.0001. **(D,E)** Fluorescence values representing single-cell expression of CD206 and CD200R, for macrophages cultured in droplets for 2 days both with and without the addition of hydrogel and with and without M2 cytokines added. “*n*” indicates number of cells measured from 1 representative donor. **(F,G)** Violin plots representing CD206 (six donors) and CD200R (five donors) expression of macrophages cultured in droplets for 2 days with either M2 stimuli, PIC hydrogel or the two combined. Marker expression was normalized to single-cell control within each donor. “*n*” indicates number of cells measured from all donors combined. Statistical significance was tested using repeated-measures one-way ANOVA with post-hoc Tukey’s test where *p < 0.05. If no significance is indicated, it was not found.

Cells encapsulated in single-cell hydrogel droplets were significantly more viable after retrieval, when compared to cells encapsulated in aqueous droplets as measured during flow cytometry (Figure 5A). Similar improvements were seen for both M1 and M2 stimulated cells (Figures 5B,C), where an overall lower viability was observed after M1 stimulation. This reduced viability is to be expected as LPS is known to induce apoptosis (Xaus et al., 2000). These findings indicate that addition of the PIC hydrogel prevents a weakened cell state similar to anoikis, allowing cells to maintain better viability, required for downstream analysis.

Phenotypical interrogation of single cells showed that addition of hydrogel resulted in an upregulation of M2 specific markers CD206 and CD200R for the majority of cells compared to the control (Figures 5D,E). For CD206 the degree of upregulation was similar to addition of M2 cytokines, whilst for CD200R the effect was even more potent. Combining both M2 cytokines and hydrogel in the droplets resulted in the highest increase of fluorescence for both markers. These experiments were repeated for six donors. To check the biological reproducibility, and to compensate for donor-donor variation, the median fluorescent intensities of each condition were compared as normalized to the control population (Figures 5F,G). This again confirmed that both the addition of hydrogel as well as M2 cytokines resulted in an upregulation of CD206 and CD200R expression, although especially for CD200R this was observed with a considerable amount of variation, where two donors did not respond at all. When the M2 cytokines and hydrogel were combined, this again resulted in an even further and more robust upregulation of marker expression for all donors. These results demonstrate that incubating single macrophages in hydrogel droplets enhances cytokine induced M2 polarization. This effect has been shown previously in bulk cultures (Cha et al., 2017) but never before for single cells.

### GRGDS Functionalization of Hydrogel Droplets Does Not Affect Macrophage Polarization and Viability but Changes Morphology

**FIGURE 6:**
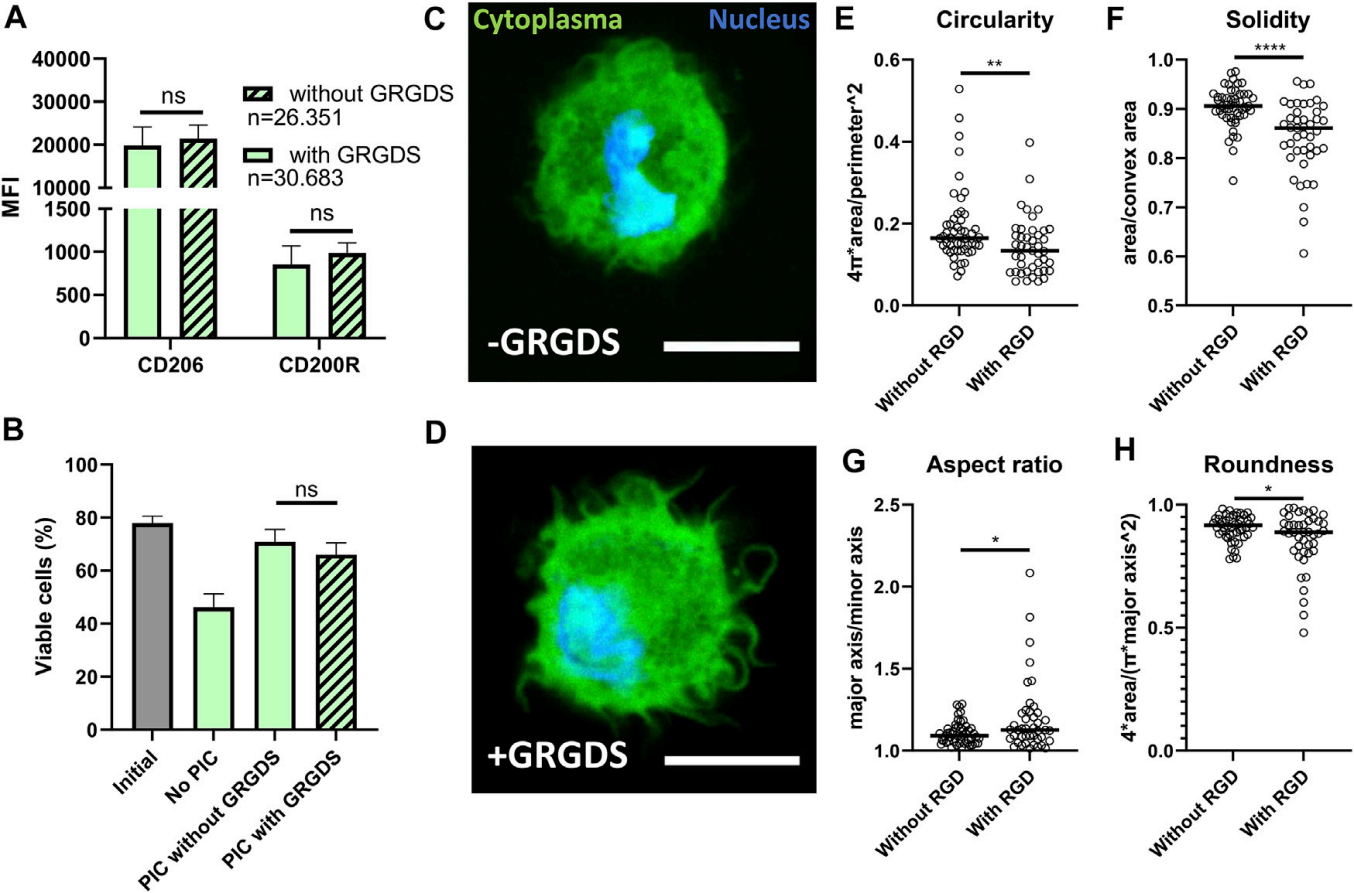
Effects of RGD functionalization of PIC hydrogels on droplet encapsulated Macrophages. **(A)** MFI representing expression of CD206 and CD200R on macrophages cultured in hydrogel droplets with and without GRGDS fragments for 2 days. Individual data points represent MFI of five different donors. “*n*” indicates number of cells measured from all donors combined. **(B)** Percentage of viable cells after 2-day culture in droplets without PIC hydrogel, with PIC and with PIC functionalized with GRGDS molecules compared to the initial viability before encapsulation. Data represents mean of five independent donors. **(C,D)** confocal images of macrophages (cytoplasma = green, nucleus = blue), in hydrogel droplets both with and without GRGDS molecules incorporated. Images were captured at ×63 magnification, scale bare represents 10 μm. **(E–H)** Circularity, Solidity, Aspect ratio and Roundness, respectively, as calculated from area, convex area, perimeter, major and minor axis derived from 93 confocal images from two different donors (without RGD; *n* = 50, With RGD; *n* = 43) where the black line indicates median. With *p < 0.05 **p < 0.005 and ****p < 0.0001.

Next, we investigated whether GRGDS functionalization of the PIC hydrogel could further positively enhance polarization because of increased adherence. GRGDS motifs are known to be the amino acid sequences most commonly responsible for cell adhesion to extracellular matrix (Ruoslahti, 1996) and have been shown to affect macrophage functions (Sridharan et al., 2015). However, in our experiments GRGDS functionalization of hydrogel in droplet culture did not result in a significant difference in M2 polarization based on CD206 and CD200R expression (Figure 6A). Additionally, the presence of GRGDS did not affect the end point viability measurement (Figure 6B), even though this effect has been observed in bone marrow stromal cells (Karoubi et al., 2009). To verify if GRGDS improved cell adherence, confocal images were captured of cells in hydrogel droplets, both with and without GRGDS molecules, to analyze changes in morphology (Figures 6C,D and Supplementary Figure 3). Although this approach is not high throughput as the droplet production and flow cytometry parts of this research, we could clearly observe that cells exhibited more distinct protrusions when GRGDS was present. To quantify changes in cell morphology four parameters were calculated from binary cell images (Supplementary Figure 1A) using ImageJ. Circularity (Figure 6E and Eq. 1) and Solidity (Figure 6F and Eq. 2) to describe the degree of cellular protrusions. Aspect ratio (Figure 6G and Eq. 3) and roundness (Figure 6H and Eq. 4) described the elongation of cells. Analysis showed that primarily the Circularity and Solidity were significantly different in GRDGS+ droplets, indicating more protrusions on the cell surface. The Aspect ratio and Roundness changed significantly indicating that GRGDS+ encapsulated cells were slightly more elongated, although this latter effect was mostly observed in several outliers.

Although cell shape was significantly altered, it did not represent the degree of morphological change as has been seen in bulk cultures for PIC (Liu et al., 2019). The degree of macrophage elongation as was shown in literature to affect M2 polarization was thus not reached in our droplet platform (McWhorter et al., 2013, 2015). This can explain the fact that we do not observe a difference in M2 related marker expression when PIC is used with or without GRGDS (Figure 6A). It is likely that, even though adherence occurs, the macrophages do not experience enough mechanical resistance in these small volumes of hydrogel to obtain a sufficient elongated state.

### Detection of Single-Cell Secretion Combined With Membrane Marker Expression Allows for the Detection of Cellular Heterogeneity

The presented single-cell data emphasize that cellular interactions and hydrogels are very potent modulators of macrophage phenotype. To probe how these phenotypical changes manifest themselves at the functional level, we incorporated the detection of secreted tumor necrosis factor-alpha (TNFα) molecules into the single-cell hydrogel platform. TNFα is an important pro-inflammatory cytokine secreted by macrophages, which plays a role in immune homeostasis (Parameswaran and Patial, 2010). Secreted cytokines were captured by pre-labeling macrophages with cytokine-specific antibodies that are anchored onto the cell membrane. Subsequently, after cell retrieval the cytokines can be detected as cell membrane expressed markers (Supplementary Figure 4A). This technique is especially suitable for droplet-encapsulated cells as secreted cytokines will not be able to bind neighboring cells, thereby avoiding false positives (Supplementary Figure 4B and Chokkalingam et al., 2013; Charles et al., 2020).

**FIGURE 7:**
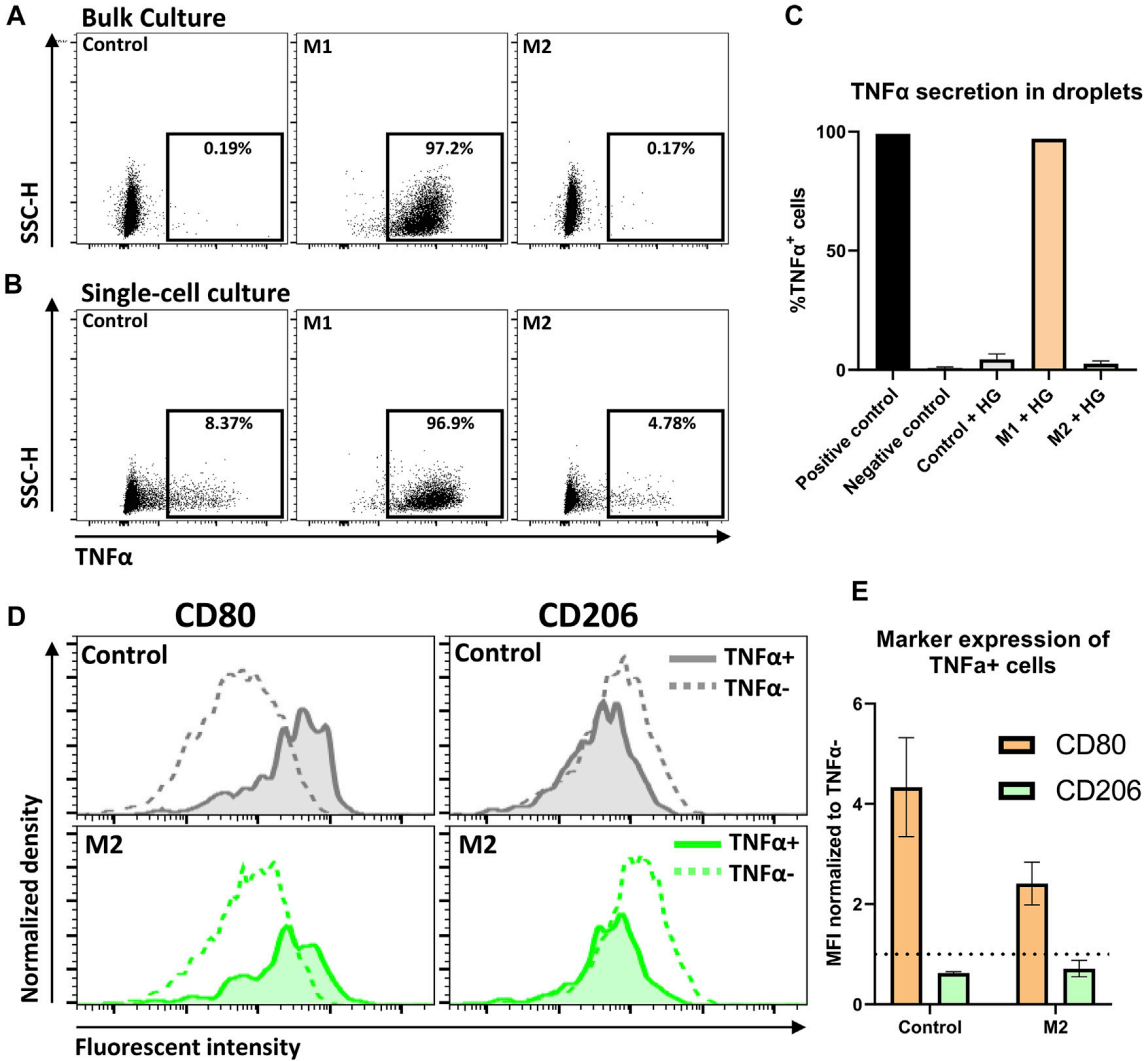
Detection of TNFα secretion by single-cell polarized macrophages. **(A,B)** Dot plots representing TNFα secretion of Control, M1 and M2 stimulated macrophages in bulk and droplets, respectively. Gating for TNFα was based on a positive control. **(C)** expression of TNFα on macrophages after 2-day culture in hydrogel droplets. Results represent mean of Control: *n* = 3 donors, M1: *n* = 2 donors and M2: *n* = 3 donors and negative control: *n* = 3, positive gate is based on positive control. **(D)** Histograms comparing expression of markers for M1 (CD80) and M2 (CD206) polarization between TNFα− cells and TNFα+ cells. Histograms show control and M2 cells from one donor cultured for 2 days in hydrogel droplets. **(E)** Bar graph comparing CD80 and CD206 expression between TNFα− and TNFα+ cells from hydrogel droplet cultured control and M2 cells. Bars represent MFI of TNFα+ population normalized to TNFα− population for *n* = 3 donors.

Bulk experiments showed that close to 100% of M1 stimulated cells were TNFα positive (TNFα+), whereas the control condition and M2 stimulated condition resulted in negligible TNFα secretion (Figure 7A). These observations are fully in line with expectations based on literature (Duque and Descoteaux, 2014; Spiller et al., 2014). Similarly, in hydrogel-droplets, all M1 stimulated cells actively produced TNFα. Strikingly, in droplets, both the M2 stimulated and control conditions showed a small percentage of TNFα+ cells (Figures 7B,C). Closer examination of these TNFα+ cells revealed an increased CD80 and decreased CD206 expression compared to the TNFα negative (TNFα−) population, indicating that these TNFα producers have a more M1-like phenotype compared to the rest of the M2-induced population (Figure 7D). This observation holds true for both the control macrophages and the M2 stimulated macrophages in three donors from independent experiments (Figure 7E), signifying the presence of persistent heterogeneity when cell-cell interactions are absent. This finding is supported by the fact that such populations could not be found in the bulk experiments. A similar heterogenous macrophage behavior was previously reported for TNFα and other cytokines in a microwell-based assay (Xue et al., 2015). Those results were in response to pro-inflammatory stimulation with LPS. Here we show however, that such behavior can arise even when cells are not stimulated or can persevere even when cells are stimulated with anti-inflammatory cytokines. Additionally, to the best of our knowledge this is the first time that such secretion behavior is observed in combination with altered phenotypical markers. Taken together, this multi-parametric analysis combined with single-cell culture holds great potential for the preservation and detection of heterogeneity in macrophage populations.

## Conclusion

In this study, droplet-based microfluidics was applied to establish a workflow to combine single-cell culture of adherent cells with single-cell endpoint measurements, in order to maintain and discover population heterogeneity. We have achieved a versatile platform that can be applied to study polarization of individual macrophages in a high-throughput fashion and allows insights into how cell-cell communication affects the degree of polarization. Additionally, the incorporation of the thermo-reversible PIC hydrogel proved to make the platform more robust and enabled potential applications for other adherent cell types. The ability to measure membrane markers and secreted cytokines in a multi-parameter fashion allowed for the detection of previously unidentified subset of macrophages that maintained a more M1-like phenotype even when being stimulated with M2 stimuli. This combination of single-cell functional and phenotypical read-out with single-cell culture holds promise for a straightforward and easily accessible tool in the discovery of novel immunological cell populations.

## Data Availability

The raw data supporting the conclusions of this article will be made available by the authors, without undue reservation.
