# Supplementary material for: Probing Single-Cell Macrophage Polarization and Heterogeneity Using Thermo-Reversible Hydrogels in Droplet-Based Microfluidics
Source: Front Bioeng Biotechnol. 2021 Oct 14;9:715408. doi: 10.3389/fbioe.2021.715408 (PMC8552120; doi:10.3389/fbioe.2021.715408)
Supplement: Supplementary file 1 [file DataSheet1.PDF]

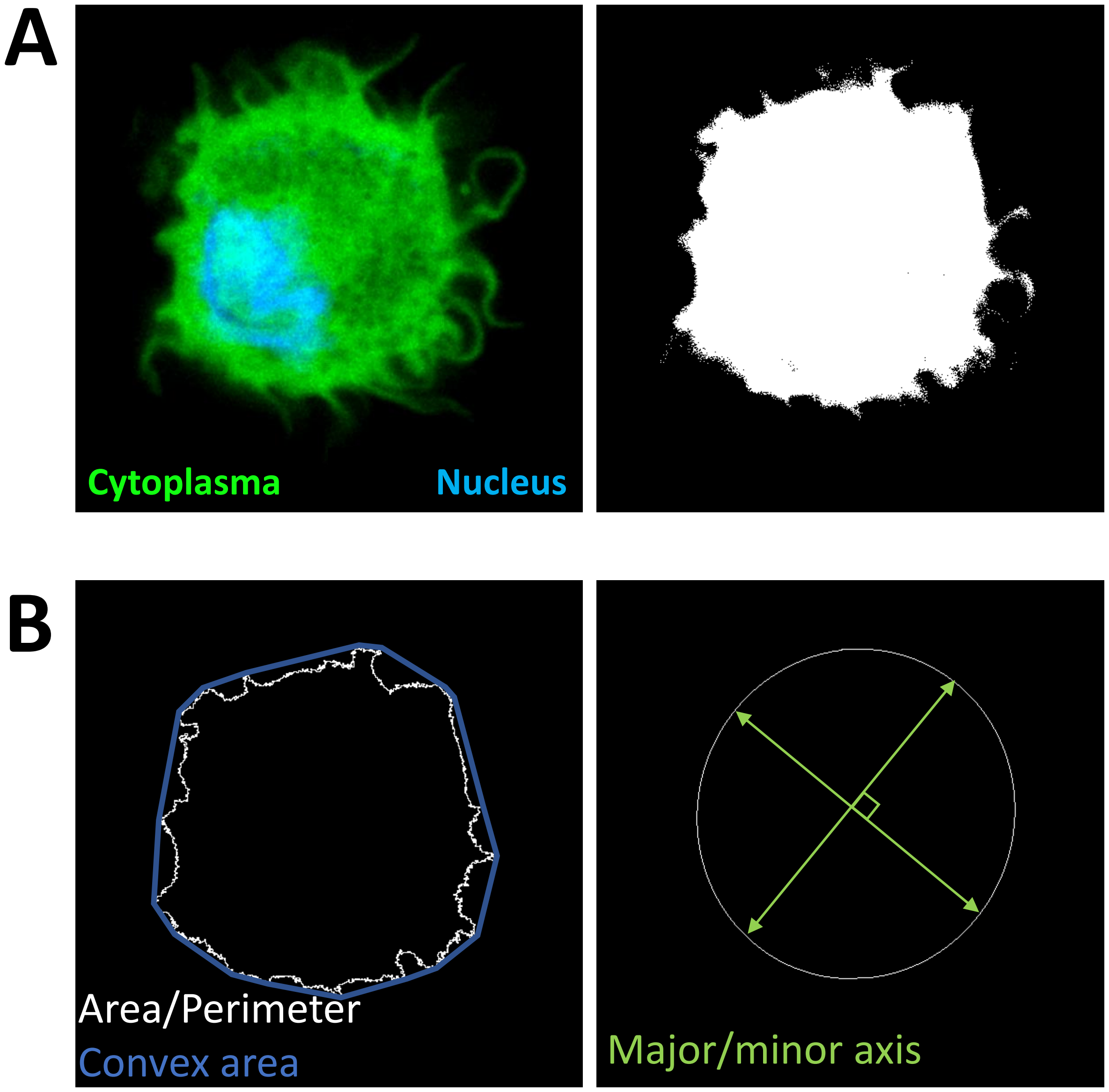

Supplementary figure 1: Analysis of macrophage morphology in droplets using ImageJ A) Creation of binary mask based on confocal images of macrophages in hydrogel. B) Dimensional measures as performed on binary masks.

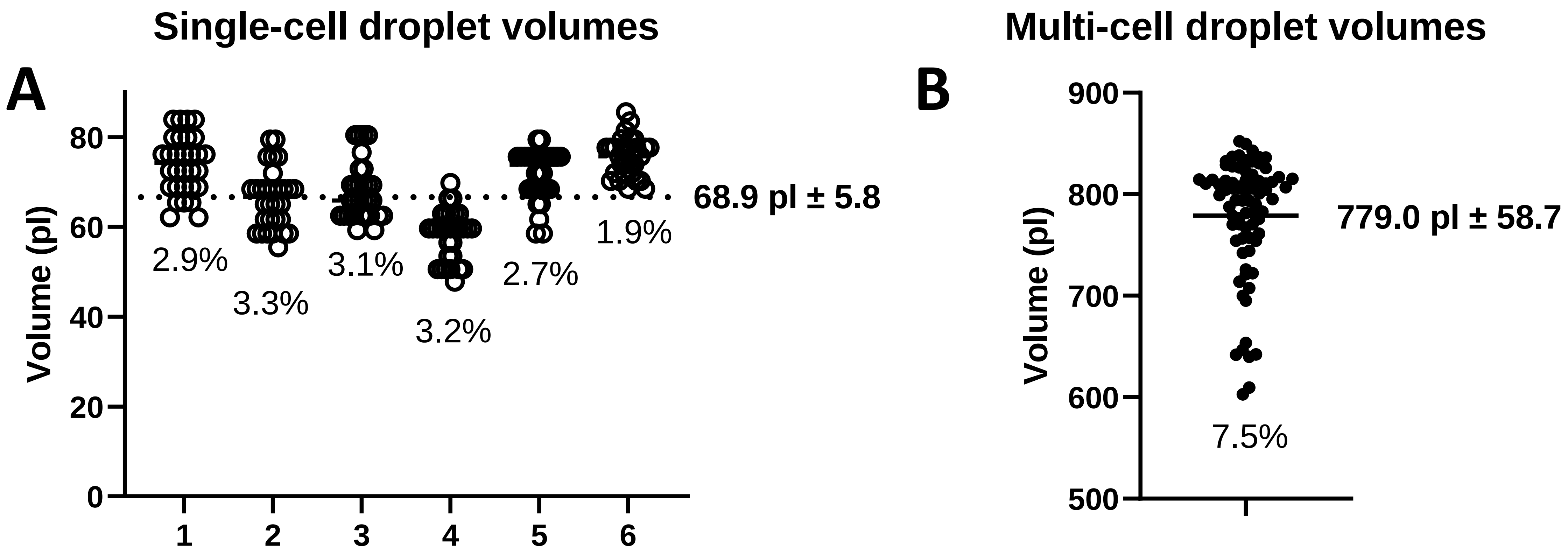

Supplementary figure 2: A) Volume of n=30 droplets from 6 randomly picked experiments created using 30 µm channel height. Volumes are calculated from diameter measured using ImageJ. Coefficient of variance (annotated bellow each event clusters) is calculated for each experiment. Average droplets sizes is 68,9 pl with a standard deviation of 5,8 pl. B) Volume of n=77 droplets created using 70 µm channel height as calculated using ImageJ. Average droplet size is 779,0 pl with a standard deviation of 58,7 pl. coefficient of variance is 7,5%.

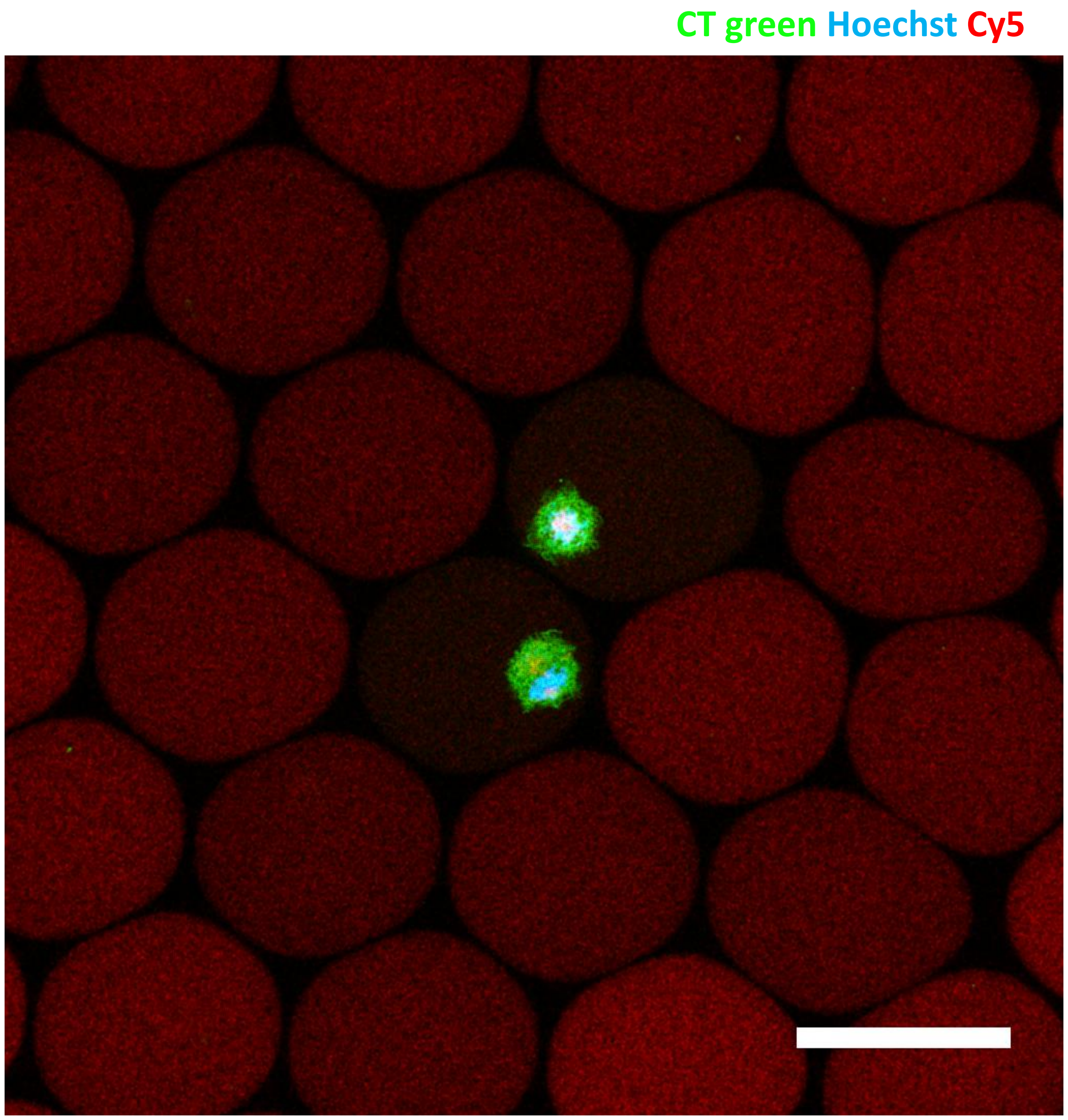

Supplementary figure 3: Overview of multiple hydrogel droplets (red) with GRGDS at 10x magnification with two droplets containing macrophages (green = cytoplasm, blue = nucleus), scale bar represents 50 µm.

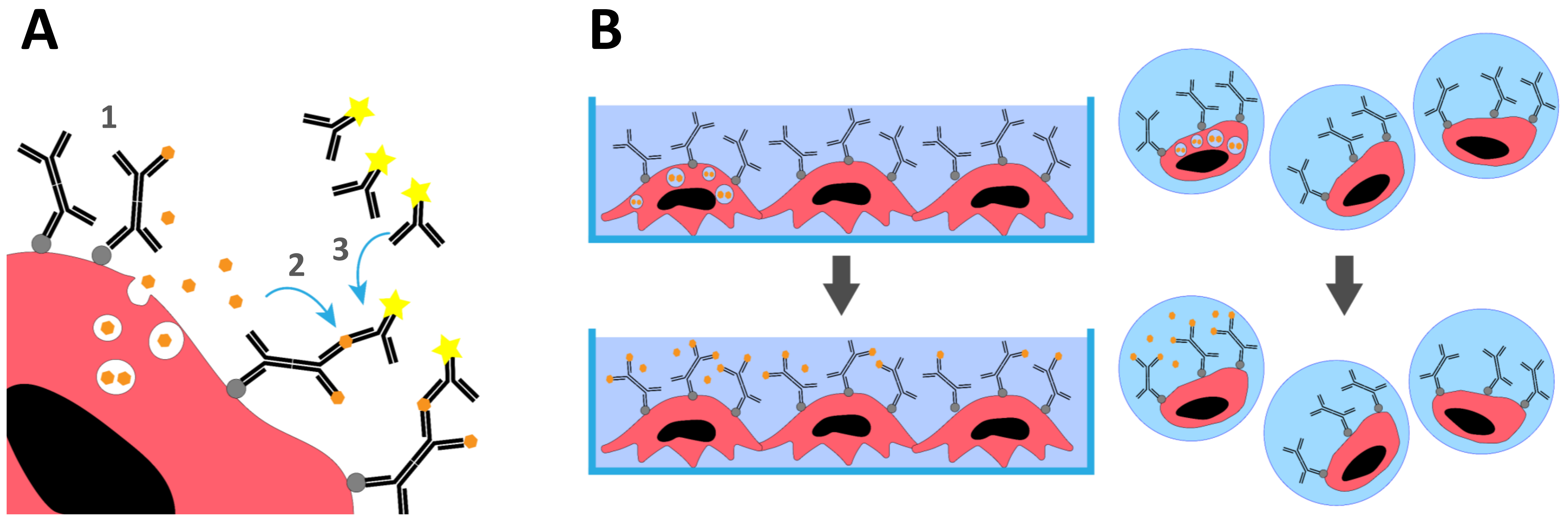

Supplementary figure 4: Illustration of working principle of the cytokine detection assay. A) 1. Cells are coated with double sided antibodies specific for TNFα cytokines (orange), 2. secreted TNFα binds to antibody coating, 3. bound TNFα can be detected by staining with fluorescent antibodies (yellow). B) Difference between cytokine detection in bulk and in droplets. In bulk TNFα (orange) from producers can bind a-specifically to non-producers creating false positives. In droplets TNFα is confined in the droplets preventing false positives ensuring true single-cell detection.
